# Directional Migration of Recirculating Lymphocytes through Lymph Nodes via Random Walks

**DOI:** 10.1371/journal.pone.0045262

**Published:** 2012-09-20

**Authors:** Niclas Thomas, Lenka Matejovicova, Wichat Srikusalanukul, John Shawe-Taylor, Benny Chain

**Affiliations:** 1 CoMPLEX, UCL, Gower Street, London, United Kingdom; 2 John Curtin School of Medical Research, Australian National University, Canberra, Australia; 3 Department of Computer Science, UCL, Gower Street, London, United Kingdom; 4 Division of Infection and Immunity, London, United Kingdom; University of Lyon, France

## Abstract

Naive T lymphocytes exhibit extensive antigen-independent recirculation between blood and lymph nodes, where they may encounter dendritic cells carrying cognate antigen. We examine how long different T cells may spend in an individual lymph node by examining data from long term cannulation of blood and efferent lymphatics of a single lymph node in the sheep. We determine empirically the distribution of transit times of migrating T cells by applying the Least Absolute Shrinkage & Selection Operator (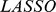) or regularised 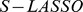 to fit experimental data describing the proportion of labelled infused cells in blood and efferent lymphatics over time. The optimal inferred solution reveals a distribution with high variance and strong skew. The mode transit time is typically between 10 and 20 hours, but a significant number of cells spend more than 70 hours before exiting. We complement the empirical machine learning based approach by modelling lymphocyte passage through the lymph node 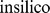. On the basis of previous two photon analysis of lymphocyte movement, we optimised distributions which describe the transit times (first passage times) of discrete one dimensional and continuous (Brownian) three dimensional random walks with drift. The optimal fit is obtained when drift is small, i.e. the ratio of probabilities of migrating forward and backward within the node is close to one. These distributions are qualitatively similar to the inferred empirical distribution, with high variance and strong skew. In contrast, an optimised normal distribution of transit times (symmetrical around mean) fitted the data poorly. The results demonstrate that the rapid recirculation of lymphocytes observed at a macro level is compatible with predominantly randomised movement within lymph nodes, and significant probabilities of long transit times. We discuss how this pattern of migration may contribute to facilitating interactions between low frequency T cells and antigen presenting cells carrying cognate antigen.

## Introduction

The anatomy of the immune system is unusual in that a number of its cellular components are motile. Antigen independent recirculation is a characteristic feature of vertebrate, and especially mammalian, adaptive immune systems, allowing optimum opportunities for cells to interact. In particular, recirculation of the lymphocyte pool is necessary so that rare, antigen-specific T lymphocytes have the best chance to encounter a dendritic cell (DC) carrying their cognate antigen.

Mackay et al. [1] describe two major pathways of recirculation in sheep, depending on the function and phenotype of the T lymphocyte. The vast majority of cells arrive at the LN via high endothelial venules (HEVs) and depart via the efferent lymphatics. Unless the T lymphocyte encounters an antigen specific for its receptor, the T lymphocyte will exit the LN via the efferent lymph vessels which drain into the principal lymphatic vessel, the thoracic duct, and continue their recirculation by re-entering blood flow. An alternative route exists, namely departing blood at peripheral vascular beds, especially at sites of inflammation and reaching the LN via the afferent lymphatics. Most lymphocytes in afferent lymphatics are of effector phenotype, whereas naive (or central memory) predominate in efferent lymphatics. Antigen-presenting cells such as DCs carry antigen from surrounding tissue via afferent lymphatic vessels to the LN, where the antigen is presented to T cells. Since infection is often local, DC migration together with T cell recirculation maximises the chance that a naive T lymphocyte will locate its cognate antigen.

Within the past decade, it has become possible to observe individual labelled lymphocytes moving within lymph nodes using two photon microscopy. In contrast to the directional flow of cells seen at a macro level, T cell movement within the lymph node appears to be predominantly random [2], [3] with no clear evidence of directionality. However, the measurements of individual lymphocytes obtained by microscopy are typically only over short times and distances, since the cells migrate rapidly outside the field of view. A robust study of lymphocyte migration therefore needs to bridge the microscopic and macroscopic view points. A number of previous studies have extended the timescale of the microscopy observations using 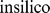 modelling. Even prior to the introduction of two photon technology for tracking lymphocyte movement, Stekel [4], [5] used differential equations to model three compartments of T cell recirculation (blood, spleen and the lymphatic compartment), and used this to derive a time course for the appearance of T lymphocytes in thoracic duct blood. Stekel linked the macroscopic properties of his model to the microanatomy of the lymph node, by proposing that transit times through lymph nodes are determined by adhesive interactions with dendritic cells which slow T cell migration [5]. Beltman et al. [3] combined two-photon microscopy imaging of the *in vivo* LN with a cellular-Potts based model of T lymphocyte migration to determine the effects of the topology of a LN on the migratory characteristics of T lymphocytes. In bestowing the 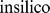 T lymphocytes with a preferred direction of motion which corresponds to their recent displacement, T lymphocytes exhibited `persistent motion on a short timescale, random motion on a long timescale, and large velocity fluctuations with apparent periodic pausing’. Bogle and Dunbar [6] also investigate a 3D lattice-based model to simulate T cell behaviour in the paracortex at realistic cell densities, successfully reproducing a cell motility coefficient that matches estimates made in previous studies. More recently, two studies [7], [8] have specifically investigated random walk migration models in the context of two photon data on lymphocyte movement through lymph nodes.

The studies listed above have mostly focused on obtaining long term predictions of lymphocyte migration times derived from models based on microscopic observations. In this study we adopt a complementary approach, to infer the distribution of lymphocyte transit times directly from long term observations of bulk migration through a single lymph node. Rather than impose an a priori model, we use machine learning approaches to infer this distribution from experimental data obtained by cannulating individual lymph nodes and collecting lymph over long periods. We use observations from a sheep model, since single lymph node cannulation, on which this approach is based, cannot be carried out in the rodent models explored in the previous studies. A brief analysis of this data has been published previously [9]. We further demonstrate that the inferred distribution of transit times inferred from the data is compatible with predicted distributions generated from a discrete Markov chain random walk model of migration, and the inverse Gaussian distribution which describes Brownian motion first passage times [10]. The results of our analysis provide an estimate both of mean transit time, and of the distribution of transit times observed, and suggest that random movement of lymphocytes within a lymph node is sufficient to account for bulk flow even in larger immune systems such as that of the sheep.

## Results

### Estimating the Distribution of Transit Times in Blood

**Figure 1:**
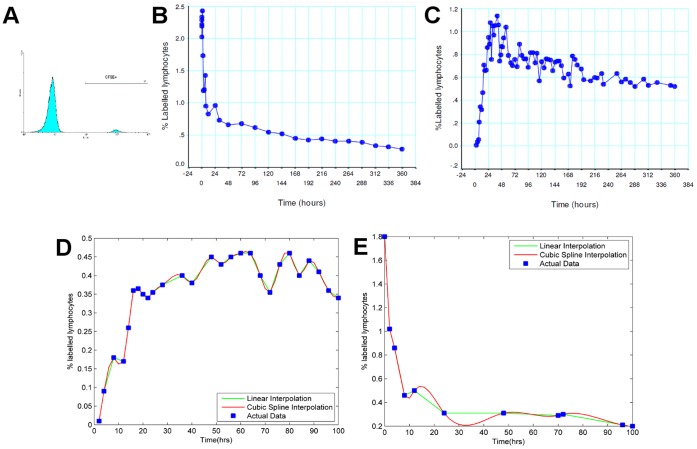
Results from a representative cannulation experiment (R705). a) Flow cytometry histogram showing CFSE positive and negative cells in lymphatic fluid. % CFSE in blood (b) and lymphatic fluid (c) at different times post infusion. (d and e) Actual and interpolated % CFSE values in blood (d) and lymphatic fluid (e) over the first 100 hours, using either linear or cubic spline interpolation to infer missing time points.

The data analysed in this study were collected from sheep in which both blood and the efferent lymphatic from prescapular (15 data sets) or popliteal (2 data sets) lymph nodes were cannulated (see Materials and Methods) for a minimum of 100 hours. An initial sample (at least 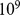) of cells in the efferent lymph (and hence primarily drawn from the recirculating naive lymphocyte population as shown in several previous publications e.g. [1]) was labelled with CFSE, and then infused back into the circulation via the carotid artery. The cells from efferent lymph were almost entirely composed of lymphocytes, of which around 70% were T lymphocytes (data not shown) [11]. After infusion of labelled lymphocytes back into the circulation, blood and lymph samples were then collected at various time intervals, and the proportion of labelled cells was analysed by flow cytometry. A representative flow cytometry histogram of CFSE expression in lymphatic lymphocytes is shown in fig. 1a and a representative timecourse (raw data) from one experiment (sheep R705) is shown in fig. 1b and 1c. Samples were collected less frequently at later time points, when the rate of change in cell numbers was smaller.

We first estimated the distribution of times that the labelled cells spent in blood. Under the assumption that lymphocytes leave blood at a rate proportional to their concentration, we fitted an exponential model of the form 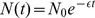 to each individual for the initial eight hours post infusion, where 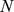 is the percentage of lymphocytes in blood at time t hours, with 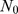 and 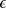 to be fitted. We thought it reasonable to fit the first eight hours post infusion as only a small proportion of total labelled cells will have exited the lymphatics and returned to the circulation before this time (see fig. 1b, and [12], [13]). The estimated mean lifetime of lymphocytes in blood in each sheep before trans-endothelial migration is given in Table 1.

**Table 1:** Mean lifetime of labeled T cells in blood.

Sheep	Mean Lifetime in Blood (hrs)
B445a	6.4
B445b	5.3
B481	9.7
B687	7.5
B857a	20.6
B857b	10.5
R153a	7.1
R153b	10.3
R401	6.4
R632	7.2
R634	10.3
R705	6.4
R797	7.2
R798	10.3
R890	7.3
Y044a	8.0
Y044b	6.3

The mean lifetime of labeled T cells in blood before trans-endothelial migration to LN, as predicted by fitting the equation 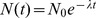.

### Calculating the Distribution of Naive T lymphocyte Migration Times in Individual Ovine LNs

The objective of our analysis was to learn the probability distribution of times which individual cells spend within the lymph node. The underlying assumption on which the analysis is based is that the number of cells which leave the lymph node at time 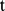 is the sum of cells entering the lymph node at time 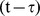, multiplied by the proportion of cells 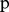 which spend time (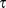) in the lymph node, summed over all the possible 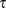. The set of p values then defines the probability distribution of times which individual cells spend within the lymph node. The number of labelled cells entering the lymph node at time t was assumed to be proportional to the proportion of labelled cells in blood at time t, while the number leaving at time t was assumed to be proportional to the proportion of labelled cells in lymph. Since the frequency of sampling was less at later time points, missing data points were estimated using either linear or cubic spline interpolation (example shown in fig. 1d and 1e). Both blood and lymph time profiles were relatively smooth at these late time points, making the data less sensitive to alternative interpolation methods. The probability distributions obtained using either interpolation method were similar (see fig. S1), and only the results obtained using linear interpolation are shown below.

**Figure 2:**
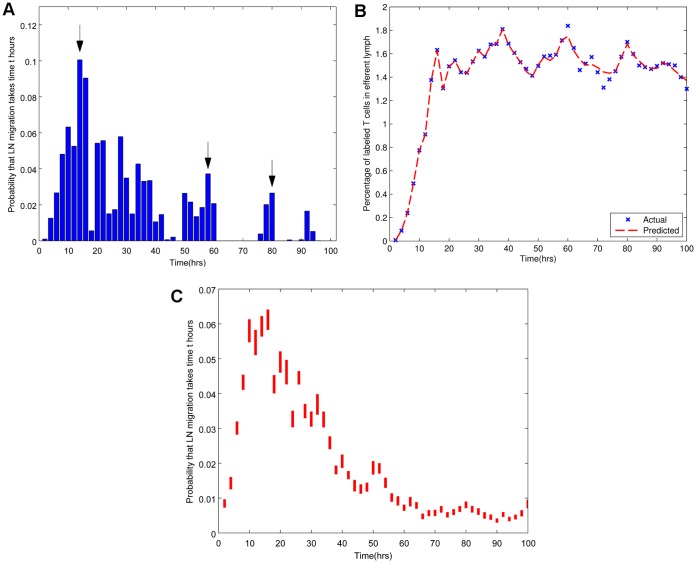
Inferred probability distribution of migration times 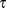 and corresponding predicted efflux profiles. The distribution of migration times calculated using 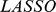 (a) and predicted and actual LN efflux based on this distribution (b) in a representative sheep (R797). Arrows indicate the possible existence of multiple peaks in the distribution of migration times. (c) Piecewise confidence intervals for individual distributions of migration times. Each bar gives a 95% confidence interval for the mean probability of migration occurring within each two-hour interval in seventeen individuals.

The set of probabilities (p) was then inferred using 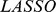 as described in Material and Methods. This approach was preferred intially, since it made no a priori assumptions about the shape of the distribution to be inferred. At the same time, it allowed the total distribution to be constrained to sum to 1, thus creating a bona fide probability distribution. A representative example of the distribution of p obtained is given in fig. 2a, along with the predicted and observed efflux in the same individual (fig. 2b). A similar analysis was carried out on data from seventeen cannulations, and the fit of the distributions we inferred from these cannulations is shown in Table 2.

**Table 2:** Average ovine LN migration times and goodness-of-fit of individual distributions to data.

Sheep	Median(hrs)	Mean(hrs)	SSE
B445a	34	40.1	0.00079
B445b	36	44.8	0.0039
B481	36	41.9	3.4×10^−23^
B687	22	34.2	0.0039
B857a	24	30.2	0.0025
B857b	26	29.3	0.0041
R153a	26	31.4	0.016
R153b	26	26.0	0.044
R401	18	29.8	0.03
R632	26	34.4	0.23
R634	14	17.6	0.72
R705	22	24.3	0.16
R797	22	29.9	0.055
R798	22	26.1	0.104
R890	22	24.6	0.19
Y044a	26	34.3	0.017
Y044b	30	34.0	0.0028

Median and mean migration times in seventeen individual prescapular and popliteal ovine lymph nodes, as calculated by 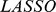.

The distributions typically showed expected migration times in the range of 17 to 45 hours (Table 2), with a median expected migration time of 30.2 hours. This data is in broad agreement with previous estimates, giving confidence to the subsequent analysis. A noticeable feature of all the distributions was that they were strongly right-skewed, with characteristically long tails, and associating significant probabilities with migration times in excess of 50 hours. Many distributions indicated the possible existence of multiple peaks in migration times (as indicated by arrows in fig. 2a). The existence of multiple peaks is also evident in fig. 2c, where for each two-hour interval, the probability for migration to occur in that interval derived from each cannulation was used to create 95% confidence intervals for the piecewise means.

### Generalising the Distributions of Naive T lymphocyte Migration Times in the Ovine LN

**Figure 3:**
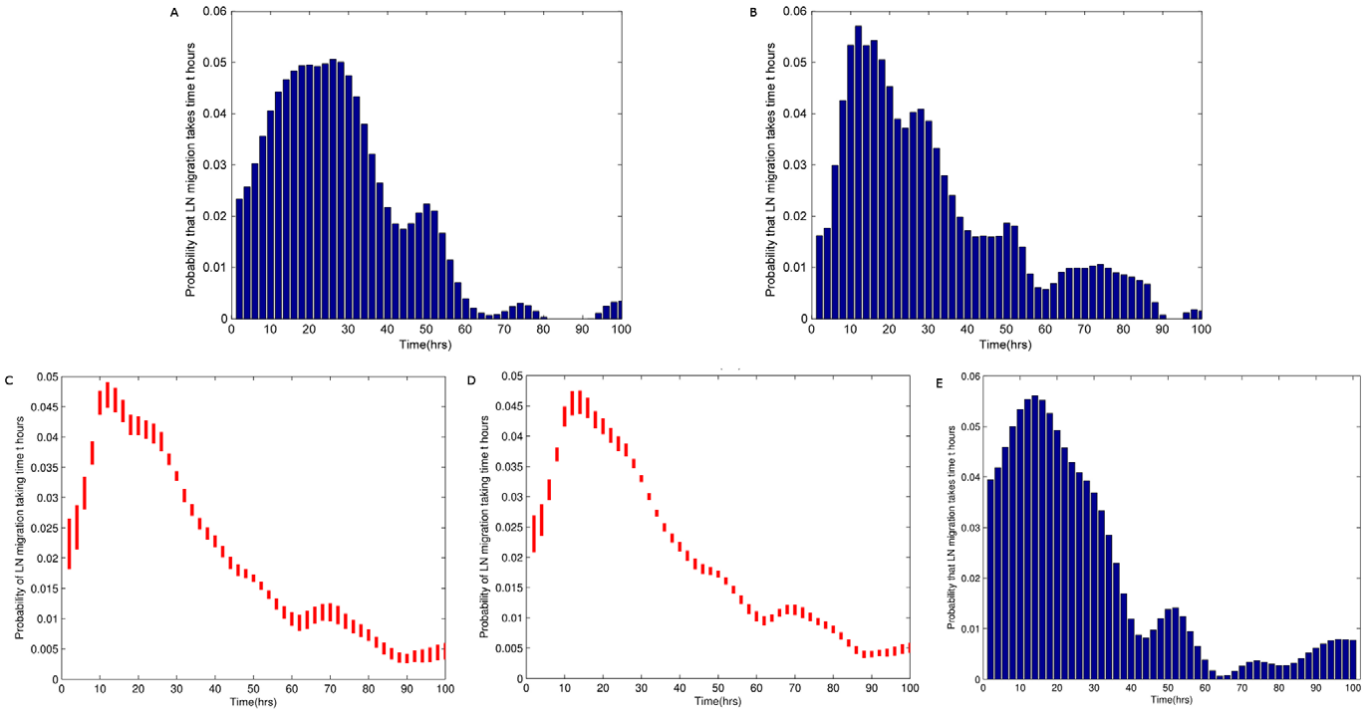
Inferred smoothed distributions of migration times obtained by 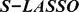 on combined data sets. Each sampled training set is optimised over 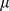 to determine the necessary extent of smoothing, and this sampling is repeated fifty times. The optimal distribution in each case is that which gives the lowest SSE on the test set out of all fifty samples. The optimal distributions shown here are based on sampling (a) three and (b) nine data sets to form the training set. (c and d) Piecewise 95% confidence intervals for optimal distributions of migration times. Each bar gives a 95% confidence interval for the mean probability of migration occurring within each two-hour interval based on fifty random samples of (c) three and (d) nine concatenated data sets to form the training set. (e) Similar analysis showing inferred smoothed distribution using CD4+ cells only (four samples).

In order to obtain a more generalised solution to our analysis, we concatenated data sets and smoothed the output using 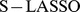 as detailed in Materials and Methods. We chose three data sets at random and implement the 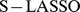 for 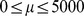 to determine the optimal 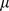 on this concatenated training set. The random sampling of data sets, concatenation and S-LASSO analysis were repeated 50 times (random sampling with replacement). The same analysis was then performed by randomly selecting and concatenating nine data sets for the training set. The distributions with the best predictive power in each case are given in fig. 3a and fig. 3b. Fig. 3c and fig. 3d show 95% confidence intervals for the piecewise probabilities obtained from the optimal distributions from each of the fifty samples.

The size of the training set and the optimal value of the smoothing parameter 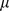 are inherently linked. An increase in training set size will usually result in a reduction in the optimal value of 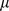, as an increase in training set size will reduce the risk of over-fitting, and hence reduce the level of smoothing necessary. Based on randomly concatenating a training set of three data sets, the optimal distribution overall was given by an optimal value of 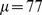, and a corresponding average sum of squared error (SSE) per data set in the test set of 0.61. The average value of 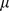 over the optimal 50 distributions was 52.62. Forming a concatenated training set of nine data sets, we found the the optimal value of 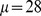, and the corresponding average SSE per data set in the test set was 0.66. The average value of 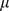 over the optimal 50 distributions was 24.5. Importantly, both analyses retain the long tail of higher transit times observed in most of the individual profiles, and also the presence of multiple peaks. Thus, these features are likely to represent true features of the underlying system, rather than computational artifacts arising from over fitting. To provide further statistical support for the existence of a long tailed distribution, we fitted, via 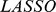, distributions where the last *n* parameters of the distribution were constrained to zero, thereby incrementally restricting a distribution with longer tails. As we increased *n*, we found the total SSE on a test set of 8 randomly chosen datasets increased, and increased at a faster rate than by constraining a random set of *n* parameters (data not shown), indicating that the long tails observed in the original distributions were a key characteristic.

An additional possible confounder was the possibility that the distribution seen arose from the combination of two or more populations of cells with very different migration properties (although previous studies have clearly demonstrated that almost all T lymphocytes in efferent lymph are naive, rather than memory phenotype [1]). Therefore we additionally stained for CD4 expression in a sample (n = 4) of sheep, and calculated probability distributions for the CD4 CFSE+ cells specifically (fig. 3e). The distribution showed similar characteristics to the overall unfractionated population.

### The Relationship between the Empirical Probability Distributions and Random Walk Models of T Lymphocyte Migration

**Figure 4:**
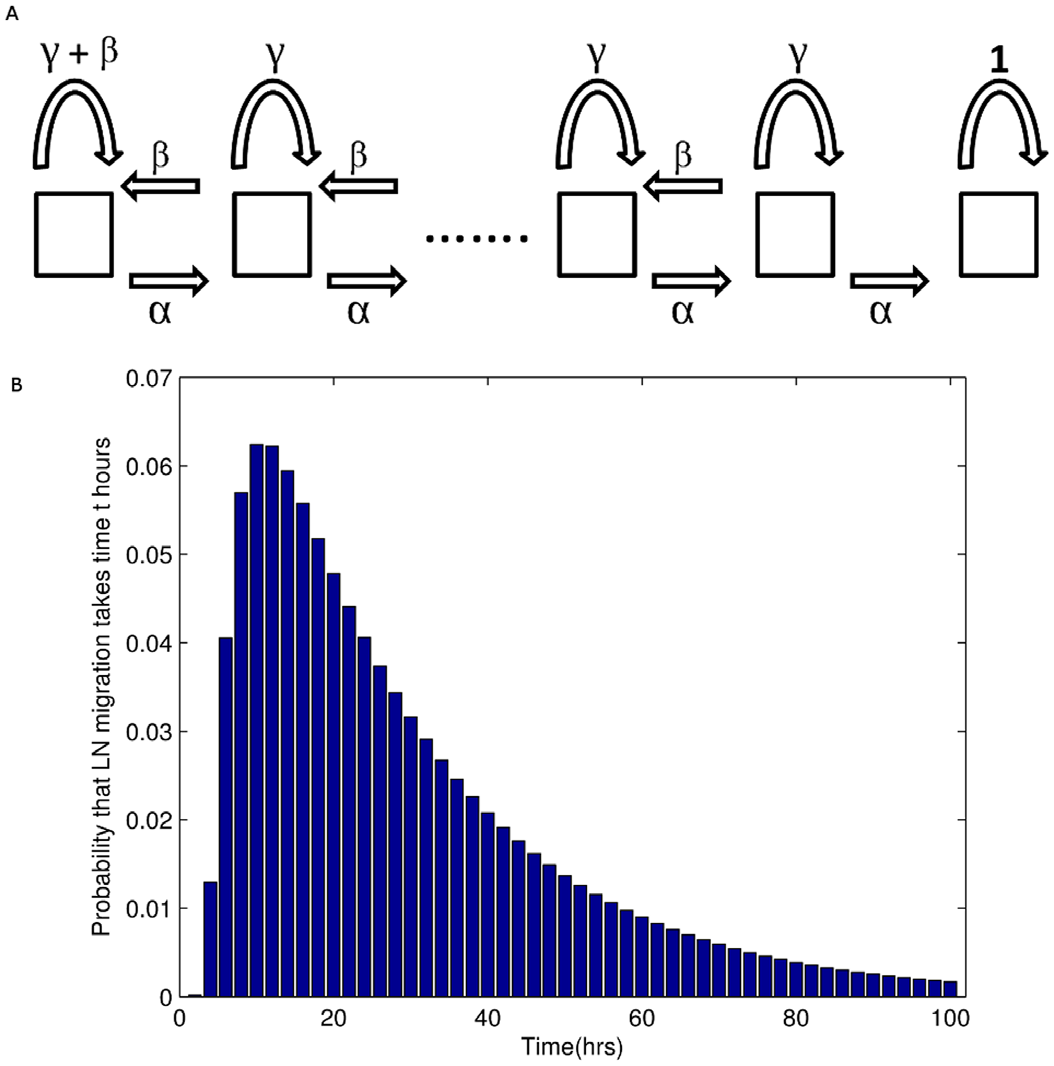
Discrete Markov chain random walk model of lymphocyte migration. a) Schematic of Markov chain model. In each time step, a cell may move to the next vertex with probability 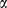, the previous vertex with probability 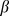 or remain at the same vertex with probability 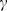. b) The optimal probability distribution of migration times 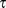 as found by the model shown in a). Parameter values are 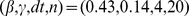. E(t)  = 28.0 hrs.

The distribution of transit times obtained above requires inference of a whole set of parameters 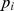, 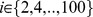. We wished to see whether the observed data could be fitted as well by using a priori distributions with smaller number of parameters. In light of the previous work exploring random walk models (e.g. [7], [8]), we first examined whether a simplified Markov chain random walk model of migration would capture the characteristic shape of transit times inferred above. We initially used a discrete one dimensional model, which captured movement along the dimension between lymphocyte entry at the HEVs and exit via the medulla into the efferent lymphatic. This model is similar to the one dimenional model analysed by Stekel [5]. However, migration perpendicular to the dimension of flow was approximately captured by allowing a probability that the cell stays on the same vertex at each time point (fig. 4a). Since this parameter is adapted to best fit the data, we hypothesised that our 1-dimensional model would capture some of the influence of the other dimensions and of the geometry of the lymph node, although we recognise that this will introduce some errors compared to a full three dimensional migration model (see below).

We calculated the probability distribution of times taken to travel from the first to the last vertex (first passage times). The model is governed by four parameters, as shown in fig. 4a, and in 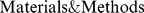. We initially optimised over 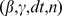 for 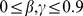 to 1 decimal place, 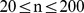 vertices and 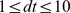 minutes. The optimal parameter set obtained from this analysis gave 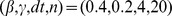. We then fixed 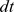 and *n* to these optimal values, and optimised 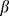 and 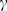 to 2 decimal places. Each pair of these parameter values gives a corresponding distribution of migration times, and predictions based on this corresponding distribution give a corresponding SSE over the test set. The distribution that gave the lowest SSE over the test set is shown in fig. 4b.

**Figure 5:**
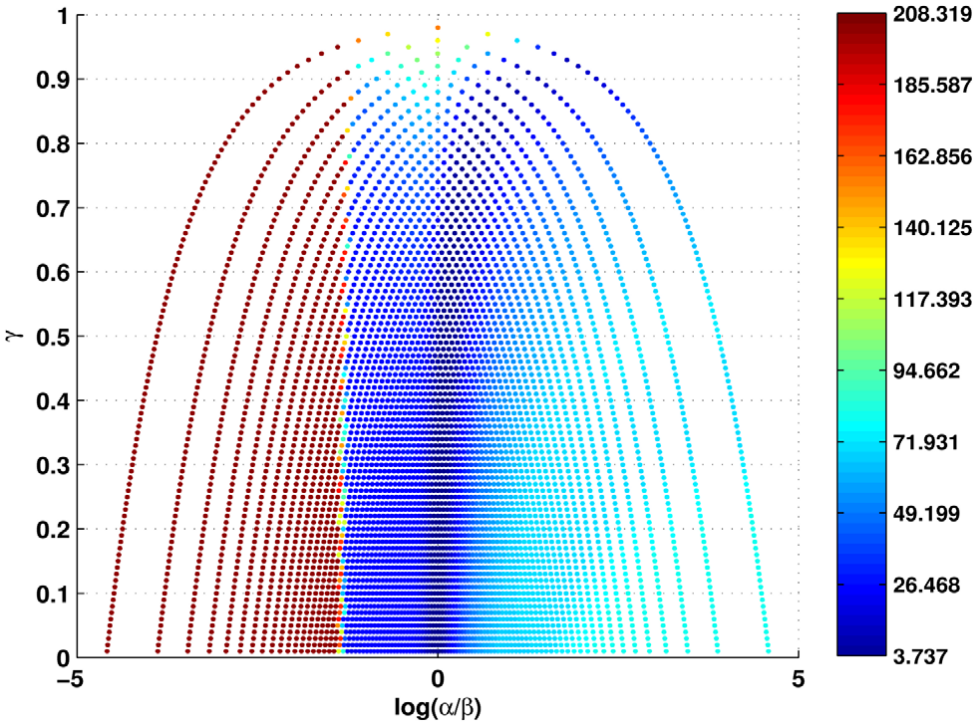
The influence of directional parameters (drift) on the the model shown in fig. 4a. The heat map shows the goodness-of-fit as 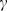 (the probability of remaining at a node) (y axis) and 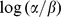 (a measure of directional movement)(x axis) are varied. Red regions represent a high SSE and blue regions a low SSE at each point in the parameter space. Best fits clearly occur when 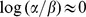, (i.e. 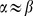), indicating quasi-undirected T cell migration within the LN.

A heat map showing 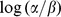 vs. 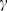 and corresponding SSE is given in fig. 5, where areas of red represent a high SSE (bad fit) and areas of blue represent a low RSS (good fit). Fig. 5 shows that a clear relationship exists between the parameters 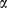 and 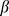. Minimal SSE over a range of 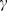 values occurs when 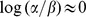 (i.e. 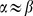). In other words, optimal fit to the experimental data occurs when the probability of forward and backward movement are approximately equal: a random walk without drift. The net flow of lymphocytes through this model is therefore driven not by directional migration, but only by the fact that entry and exit from the lymph node are unidirectional.

**Figure 6:**
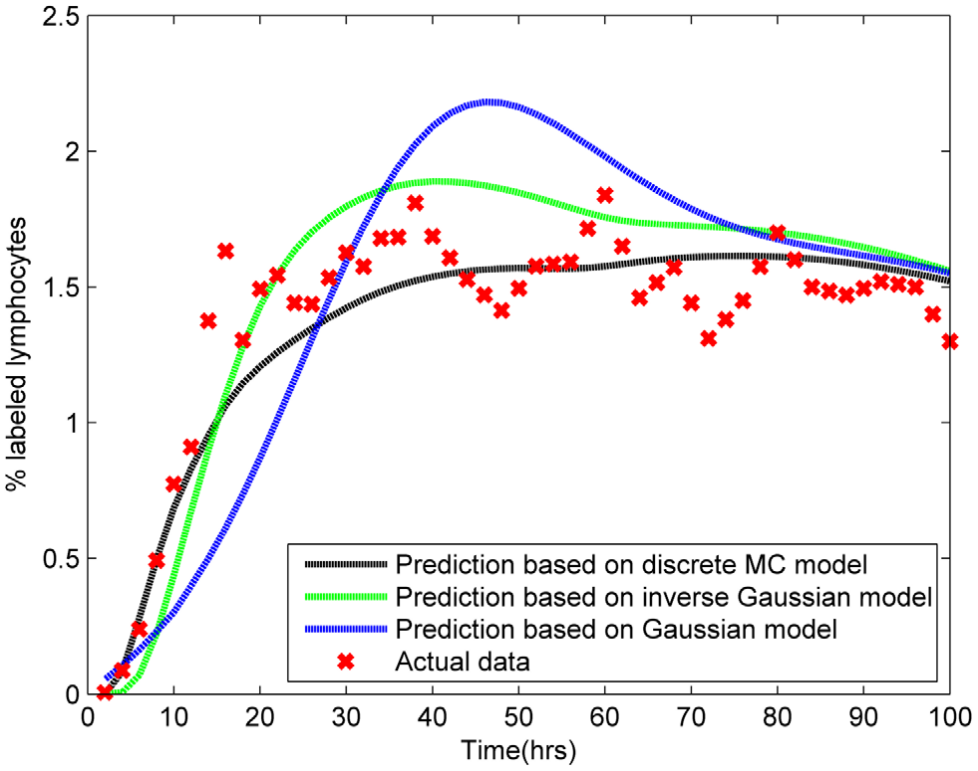
A comparison of the predictive accuracy of different transit time probability distributions. The parameters 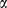, 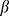 and 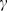 for a discrete random walk model (fig. 4a), the two parameters describing the inverse Gaussian distribution or the normal distributions were optimised as described in the text. The optimum probability distributions were then used to predict efflux. The results for a representative experiment (R797) are shown.

The predicted efflux data, based on the optimal probability distribution shown in fig. 4b, are compared to actual efflux data for one representative experiment (fig. 6). The random walk model captures the qualitative features of LN efflux following 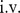 introduction of labeled lymphocytes into a sheep. The sharp rise in the percentage of labeled lymphocytes during the first six to ten hours post-infusion is followed by a peak in lymphocyte detection in efferent lymphatics at around 30 hours. This peak is then followed by a gradual decline to an equilibrium.

Since the one dimensional random walk can be considered as a discretized special case of Brownian motion, but recognising that movement in the lymph node is in three dimensions, we chose to explore the inverse Gaussian distribution, which describes the probability distribution of first passage times for Brownian motion with drift (eqn. 1) [10], [14].
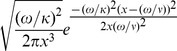
(1)


Optimising the parameters of this distribution by minimising the SSE of predicted versus observed efflux in a training set of data, and then evaluating on a test set (see Methods), we noted that the SSE decreased as the drift parameter of the distribution was decreased, with optimal fit being obtained at values of low positive drift (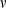  = 1).
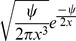
(2)


The random walk models above were compared to a directed migration model in which T cells entering at time *t* move through the lymph node together directionally, with normally distributed times. We therefore predict LN efflux by optimising the parameters 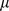 and 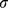 in a normal distribution of transit times over the same training set and compare SSE over the same test set.

Fig. 6 shows the predictions of lymph output based on optimised parameters for a discrete step random walk, the inverse Gaussian distribution and the normal distribution. The discrete one-dimensional random walk and the inverse Gaussian distribution give very similar predictions, and follow the shape of the data well. LN efflux inferred from normally distributed T cell migration times was a poorer predictor, showing a delay in reaching peak levels, and also a larger concentration of cells appearing at this peak.

Table 3 shows a summary of the performance of the four models discussed above, by comparing the SSE (predicted versus experimetnal efflux) obtained on the eight data sets in the test set. Each model is optimised for parameters learnt from the training set. The normally distributed model of migration times shows the worst fit to the data overall. The multimodal LASSO derived distribution, the one dimensional Markov chain model and the inverse Gaussian distribution all behave in a rather similar fashion, with no one model providing consistently the best estimate. Further data, perhaps at higher temporal resolution, will be required to distinguish these alternative models.

**Table 3:** Average ovine LN migration times and goodness-of-fit of individual distributions to data.

Sheep	SSE(LASSO)	SSE(MC)	SSE(Inverse Gaussian)	SSE(Gaussian)
B445a	1.04	1.20	1.21	0.88
B857a	8.02	12.20	5.39	19.50
B857b	1.19	1.59	0.27	0.93
R632	57.45	64.48	46.38	48.52
R634	13.07	11.98	13.23	24.23
R797	1.92	1.48	2.89	8.11
R798	2.49	1.62	3.96	7.21
Y044b	0.87	0.58	1.47	1.83

SSE on a test set of eight sheep from three different distributions.

By changing one entry in the stochastic matrix that defines the random walk stochastic matrix, we can model the steady state distribution of migrating lymphocytes within the lymph node during periods without antigenic challenge. During such periods, the number of lymphocytes leaving the LN is equal to the number entering it, since lymph node size remains constant. The stochastic matrix that defines the system becomes
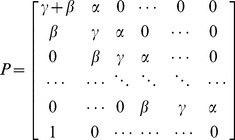
(3)


**Figure 7:**
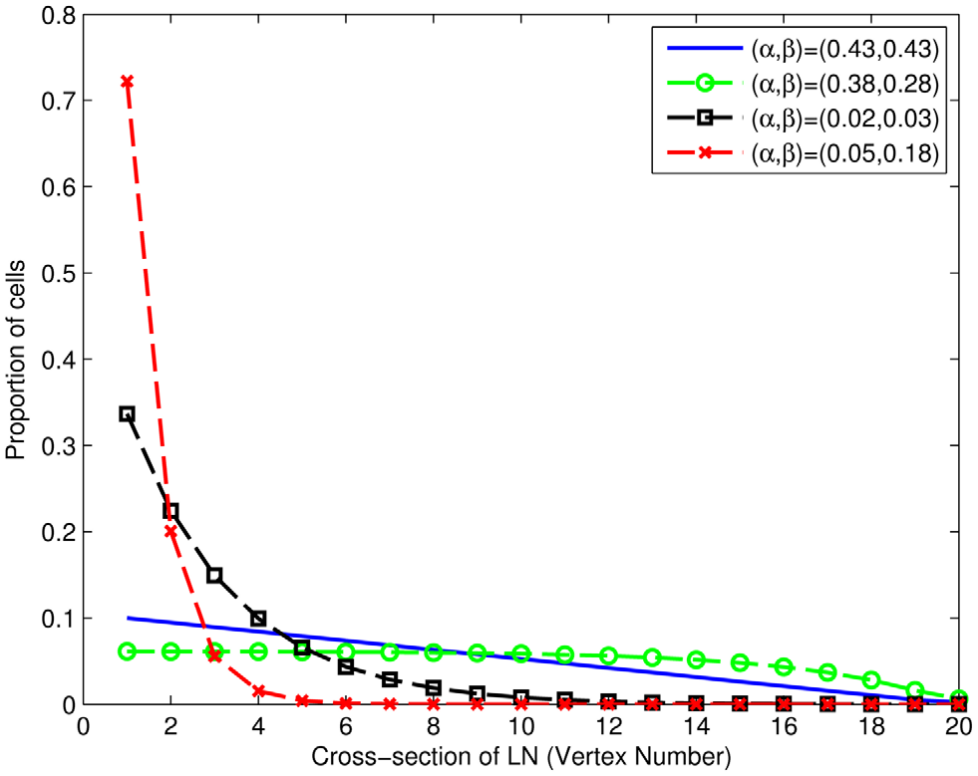
The distribution of T lymphocytes across the LN under steady-state conditions, as predicted by various parameter combinations in the random walk model. The solid blue line represents the optimal combination of parameter values, whose distribution of migration times best fits the LN efflux data. Parameter combinations which poorly predict LN efflux (dashed line with crosses and dashed line with squares) predict unrealsitic accumulation near HEVs.

The optimal parameter sets determined above (i.e. lowest RSS on test) were tested in the modified stochastic matrix under stationary conditions (i.e. steady state where entry and exit balance). Lymphocyte movement governed by this optimal parameter set (see fig. 5) predicted an accumulation of cells nearer the HEVs (vertex 1 in the model) in the lymph node and a gradual decline in cell concentration closer to the medulla region of the LN (node *n*) (fig. 7). This prediction is in agreement with experimental data [15], where T lymphocytes are observed to congregate near HEVs, which is thought to maximise chance encounters with cognate DCs. Those parameter combinations which poorly predict LN efflux (dashed line with crosses and dashed line with squares) predict far greater accumulation near HEVs, to such an extent that T cells are barely present in many other cross-sectional regions.

## Discussion

In this study we present the results of machine learning and model based analysis of lymphocyte migration data obtained from long term cannulation of sheep lymphatics. The major conclusion of the study is that the rate of migration of T cells through an individual lymph node is very heterogeneous, and is consistent with models in which individual T cells within the node move randomly from the point of entry to the point of exit. In the first part of the paper we derive a probability distribution of transit times directly from bulk migration data, using constrained least squares 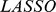. The modal transit times were observed to lie between 10 and 20 hours, while expected (mean) transit times ranged from 24 to 44 hours (Table 2). These estimates broadly agree with those published in the literature. Early studies in sheep, cannulating the efferent lymphatic of a single node [16] but using radiolabelling rather than fluorescent label, observed maximum radioactivity in the efferent lymphatics between 27 to 36 hours, and a subsequent rapid fall in radioactivity. A more recent study [17], using fluorescently labelled lymphocytes showed a much slower fall in the percentage of labelled cells, closer to that observed in the present study. The loss of radiolabel from cells may have contributed to an artificial shortening of detection times seen in the earlier studies. In rodents, where cannulation of individual lymph nodes has not been possible, most studies have measured the overall migration from blood through the lymphatic system by cannulating the thoracic duct. Ford & Simmonds [18] studied recirculation in the rat, estimating modal LN migration times to be between 16 and 18 hours, and Sprent [19] studied the mouse, with maximum percentage of cells in efferent lymphatics between 24 and 30 hours (varying in different mice). It is difficult to really compare the quantitative estimates of modal transit times between these studies, since passage to the thoracic duct may include serial passage though lymph node chains, and relatively long passage times though the draining lymphatic network. Nevertheless, there seems to be no evidence of any clear relationship between transit times and size (e.g. sheep and mice lymph nodes differ by more than three orders of magnitude). Although at first sight surprising, the observation may be understood in terms of the equation
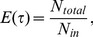
(4)


under the assumption of steady state when input and output to the node are equal, where 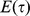 is the mean transit time, 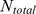 is the total number of cells in the LN and 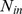 is the number of cells entering the LN per hour. Thus in larger species, a faster inflow (greater blood flow to individual lymph nodes, and hence a greater area of HEV, for example) may offset the larger volume of lymph node. The most striking outcome of the analysis of transit times using 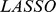 is the high variance, breadth and skewness of the inferred probability distributions. The estimated probabilities of transit times less than 2–4 hours is low, presumably reflecting the minimum time required for a lymphocyte to leave blood within a HEV, move through the lymph node and reach the efferent lymphatic. A recent study suggested that in the mouse this distance may be quite short, because T cells exit via medullary sinuses which extend deep into the T cell zone [8]. Unfortunately, similar detailed lymph node anatomy is not available in the sheep. However, the distributions (both individual, or derived by pooling data or smoothing the distributions), consistently demonstrate a long tail of transit times, with significant numbers of cells taking 70 to 100 hours to leave the lymph node. Thus, as noted by Stekel [4], [5] in his analysis of rat migration data, significant ‘mixing’ or retardation of lymphocytes occurs during passage though the node. The cellular mechanisms which might give rise to the observed distribution are discussed in more detail below.

An unexplained feature of the transit time distributions ‘learned’ from the data by the constrained regression analysis (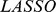) is the existence of several secondary probability modes. Possible explanations for the features could include heterogeneous entry/exit points (e.g. HEVs at different distances from the efferent lymphatics, and hence different path lengths for migration), or the presence of heterogenous populations of cells. The vast majority of T cells within the efferent lymphatics are naive cells [20] and gating on CD4 cells did not change the overall pattern of the distribution observed. However, we cannot completely rule out the possibility of further heterogeneity within the naive CD4 T cell population and further experiments will be required to distinguish these possibilities. Multimodality emerged as a robust feature of the LASSO learning algorithm, persisting in analysis of concatenated data sets, and after computational smoothing. They are unlikely to represent artifacts introduced by interpolation since different interpolation methods give rise to similar distributions. However, the multimodal distributions do not in fact provide consistently better estimates of efflux data than some of the unimodal models considered. The biological significance of this finding will therefore require further analysis.

Several previous studies have used 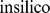 modeling to predict lymphocyte migration times across lymph nodes, based on hypotheses of lymphocyte migration behaviour. An early example [4], [5] which predated the advent of two photon imaging of lymphocytes *in vivo* proposed that lymphocytes travelling through the lymph node were slowed by competitive reversible binding to limited sites on dendritic cells via adhesion molecule interactions. Although this model accurately predicted the results of cannulation experiments in normal and lymphopoenic rats, more recent microscopic studies suggest that interactions between naive T cells and dendritic cells are very short lived [21]. Instead, the two photon data (e.g. [2], [3]) suggest that T cell migration in the lymph node is made up of a series of steps at high velocity (in the order of 10–30 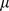
m/minute for 2–5 minutes) followed by an abrupt change of direction and another period of high velocity movement in another direction. Although T cells may show some directional behaviour in the immediate vicinity of an antigen-bearing DC [2], [22], the overall observed migration of individual T cells is largely non-directional [7], [23]. This conclusion has been supported by 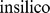 studies demonstrating that random walk models of T cell movement, coupled with information on lymph node architecture, successfully predict movement of murine lymphocytes through lymph nodes [7], [8]. In the light of this previous modelling literature, and of the two photon microscopy data, we tested some simplified random walk models of lymphocyte migration using the same set of sheep migration data. Following the studies of Stekel [5], we initially used a one dimensional discrete MC model (shown in fig. 4a), although we recognise the model fails to capture all the complexity of the three dimensional architecture of real lymph nodes. A computationally straightforward three dimensional equivalent is provided by the analysis of first passage times for Brownian motion with drift, which is described by the inverse Gaussian function [10], [14]. Interestingly, in both one dimension and three dimensions, the best fit was obtained when drift is close to zero i.e. when the relative probability of forward and backward movement is close to one. Under these conditions, the qualitative shape of the passage time distribution was similar to that inferred directly from the data, showing high variance and strong skew towards long transit times. In contrast, optimised a priori distributions with more symmetric features (random walk with strong drift, or directional migration represented by a normal distribution) were much less effective at predicting the observed migration data. Although the 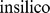 models we investigated clearly remain an over simplification, since they do not capture the details of lymph node architecture, random migration with minimal drift or direction seems to be a consistent feature emerging from the data. The recirculation of naive T cells is now widely believed to function in order to expedite the interaction between a rare antigen-specific T cell and a dendritic cell presenting its cognate antigen. Optimisation of this search strategy is therefore likely to play an important part in determining migration behaviour. Since the distribution of T cell clones through the circulation is random, a rapid and unidirectional passage of T cells across a population of dendritic cells might appear to be the optimal way of selecting antigen specific clones. Nevertheless, a number of factors contribute towards favouring a slower passage though lymph nodes. Each time T cells leave a lymph node they spend a significant time in circulation, with a median expected time of 7.3 hours (Table 2). Generally, an increased ratio of time spent within lymph nodes to time spent recirculating in blood favours the likelihood of an antigen specific interaction. However, the rate of antigen specific DC/T cell interactions is determined by the number of T cells sampled by a DC per unit time. Since a dendritic cell can interact with multiple T cells, and given a fixed distribution of sampling times [2], optimal search efficiency requires a fixed ratio of T cells to DC within a node. If one assumes that the number of dendritic cells within a node is determined a priori by the size of the area drained by the afferent lymphatic system to that node, the optimal number of T cells required at any one time to optimally service all the dendritic cells of the node will be similarly fixed. Since the mean transit time is related to the total number of recirculating cells within the lymph node (see eqn. 4), the long mean transit time observed may reflect the overall kinetics required to ensure there are sufficient T cells within the node to service the resident DC population. Interestingly, this interaction may also explain the fall in lymph node output following antigen stimulation, since antigen stimulation is accompanied by a rapid increase in numbers of lymph node DC, requiring a corresponding increase in transiting T cells within the node. This model, in which dendritic cells dictate T cell numbers, and hence T cell migration kinetics, is closely related to the model of T cell migration explored by Stekel [5], although as discussed above we do not have to posit any long term dendritic cell/T cell interaction. In conclusion, we extend previous computational analysis of lymphocyte migration to a sheep experimental model, where it was possible to follow migration through a single lymph node over several weeks. The distributions demonstrate a very heterogeneous range of lymphocyte transit times compatible with random walk models of migration within the node, in which overall bulk transit of lymphocytes is achieved by directional input and output only. The study thus adds to the emerging consensus that migration of T lymphocytes though the lymph node is predominantly random, even in larger animals such as the sheep where distances of migration are significantly larger than in the mouse. Random migration ensures good mixing of transiting lymphocytes with resident antigen presenting cells, and hence may facilitate rare antigen specific encounters.

## Materials and Methods

### Experimental Methods for Data Collection

The experimental procedures are published in more detail as part of a PhD thesis [24]. The experimental protocol was approved by The Australian National University’s Animal Ethics Committee. The experiments were carried out at the John Curtin School of Medical Research, Australian National University, Canberra, Australia. After completion of experiments, sheep were euthanized by an intravenous bolus injection of phenobarbitone solution, according to the Australian national University Animal Ethics guidelines.

#### Cannulation procedures

Merino ewes aged between three and five years were kept in metabolic cages. The cages allowed animals to lie and stand freely, and provided free access to water and food. Animals were kept caged for at least 3 days prior to surgery to allow them to accustom themselves to the experimental laboratory environments.

Sheep were anaesthestised (thiopentone sodium, 4 mg per kg animal weight) and a size 9.0 cuffed endotracheal tube was introduced into the trachea of the sheep to maintain its airway during surgery. A gas mixture of 1–3% halothane and 100% oxygen was administrated via the endotracheal tube from a Boyle’s anaesthetic apparatus. Under full anaesthesia, wool covering the operative area was removed using an animal clipper with a fine comb blade.

In each animal, data was collected from two locations, namely the external jugular vein and either the prescapular or popliteal lymh node. The popliteal lymph node, was cannulated using the operation described by Hall and Morris [25]. The operation to cannulate the prescapular lymph node has been described by Pederson and Morris [26].

The desired efferent lymphatic vessel was located and the direction of lymph flow was identified. If more than one lymphatic vessel was found, the largest one was chosen to be cannulated while the others were tied off. The efferent vessel was carefully dissected to remove all surrounding fat and tissues and ligated with 3 metric silk at the distal end of the efferent vessel as far from the node as possible. A second, loose, ligature was placed around the vessel about 1–2 cm below the first ligature to provide sufficient length for cannulation. The cannula was slowly inserted into the lymphatic vessel until its tip passed through the point of the second ligature. Then the second ligature was firmly tied. Additional ligatures were sometimes required to maintain the alignment of the cannula and ensure there were no obstructions. At the final step, the flow of lymph was checked to ensure that it was running freely before the wound was closed. Benzyl penicillin was applied to the wound. The skin was closed with Michel wound clips without any suture to muscles or aponeurosis. The external cannula was secured to the skin by sutures with 1 metric silk. Efferent lymph was allowed to drain freely under internal pressure within the lymphatic vessel.

The technique of percutaneous vascular catheterisation follows the procedure described by Seldinger [27]. It allows catheter entry into an area without an incision as a needle is used to introduce the catheter. Thus it minimises injuries to surrounding tissues. A 14-gauge needle was introduced through the wall of the external jugular vein, followed by the advanced portion of a wire guide. The needle was withdrawn while the wire guide was in place. A 16-gauge catheter was introduced into the vessel with a twist motion along the wire guide. The wire guide was removed and the catheter was secured to the skin of the sheep by multiple sutures with 1 metric silk. Normal saline solution was used to flush the catheter and blood was drawn to ensure that there was a free flow within the catheter.

General anaesthesia was normally terminated at the time of skin closure. Sheep were allowed to recover from the influence of general anaesthesia in the operating theatre. Then they were moved to metabolic cages for full recovery. The endotracheal tubes were left in place during the recovery period to prevent any possible airway aspiration and removed after post-operative evaluation. To prevent clot formation in draining lymph, a sterile solution of Ethylene Diamine Tetra Acetic acid, at a concentration of 100 mg/ml was continuously infused by a peristaltic pump and mixed with draining efferent lymph via a three way glass connector to achieve a final concentration in the lymph of approximately 2 mg/ml. A sterile mixture of 0.9% normal saline solution and heparin at a final concentration of 50 units/ml was continuously infused by a peristaltic pump at a rate of approximately 10 ml/hr through the indwelling intravenous catheter to prevent blood clot formation at the tip of the catheter.

#### Lymph collection and labelling

Animals were allowed to recover for a few days after the operation before starting an experiment. Overnight lymph (i.e. about 12–18 hours) from the cannulated efferent lymph vessel was collected in polypropylene bottles, standing in a container partly filled with ice. Lymphocytes were labelled with fluorescent dye under sterile conditions. Lymphocytes (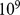 minimum) were washed with Phosphate Buffered Saline (PBS) three times. The final concentration of the lymphocyte suspension was adjusted to approximately 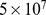 cells/ml. 5-(and-6)-carboxyfluorescein diacetate, succinimidyl ester, (CFSE), was mixed with the lymphocyte suspension at a concentration of 3–5 per 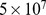 lymphocytes. and incubated in a warm bath at 37°C for 15 minutes with occasional mixing. An equal volume of cell-free lymph was added to stop the reaction. Lymphocytes were washed 3 times and resuspended in PBS. If cell viability or staining (as measured by flow cytometry) was less than 90% the experiemnt was terminated.

CFSE labelled lymphocytes (generally about 1–2×10^9^ cells) were infused back into donor animals via the indwelling intravenous catheter as a bolus within a minute followed by 10–20 ml of normal saline to flush any lymphocytes adherent to the tubing.

For each blood sample, approximately 3–5 ml of venous blood was manually drawn from the indwelling venous catheter into a 5 ml sterile plastic syringe containing 0.5 ml of 2% EDTA. Heparinised normal saline was used to flush the remaining blood in the tubing after sampling. The frequency of blood sampling was greater during the first few hours and decreased after that. In most experiments, blood sampling was undertaken at 2, 5, 10, 20, 30 minutes, and 1, 2, 3, 4, 6, 12 and 24 hours after infusion, and thereafter every 24 hours until the end of the experiment (i.e. about 7 to 12 days depending on the conditions of each individual animal and experiment.

The efferent lymph (well mixed with 2% EDTA) was allowed to drain freely and collected into 15 ml plastic containers using a fraction collector programmed to collect the lymph every 2 to 8 hours. A period of about 15–30 minutes was required for collection of each sample of efferent lymph, depending on the lymph flow rates in individual animals at the time of experiment. The volume of draining efferent lymph was recorded and the lymphocyte concentration was counted using a haemocytometer every 12–24 hours to determine the total number of lymphocytes drained from the efferent lymph during that period.

Lymphocytes from lymph samples were washed three times in PBS with 2% Bovine Serum Albumin, 2 mg/ml EDTA and 0.1% Azide (PBS/BSA/EDTA/Az) and analysed by flow cytometry. Blood samples were treated with a mixture of 0.83% ammonium chloride and 0.17 M Tris buffer (9∶1) to remove red blood cells. In some experiments, CD4 expression was measured before subsequent fixation. The cells were labelled using standard indirect immunofluorescence protocols. The monoclonal anti-CD4 antibody SBU-T1 (25.91) obtained from the Centre for Animal Biotechnology (School of Veterinary Science, The University of Melbourne, Victoria, Australia) was used as first layer, and visualised using a phycoerythrin-labelled anti-mouse immunoglobulin (Silenus Laboratories, Australia) as second layer. All flow cytometry analysis was done on a FACScan flow cytometer. Samples were gated on forward and side scatter to exclude debris and cell clumps. The number of cells analysed was approximately 20,000–50,000 for each sample. All flow cytometry studies were undertaken within a week of the cells being fixed. A final concentration of 10 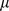
g/ml of propidium iodide was used to stain lymphocytes before and after labelling with CFSE to determine their viability.

#### Least squares regression

Sampling of blood was carried out more frequently during the first twenty-four hours of the experiment, and at every twenty-four hour interval subsequently and so we estimated labelled lymphocyte counts between late data collection points. We used both linear and cubic spline interpolation (as shown in fig. 1), and both methods gave similar probability distributions. We assume that T cells enter the lymph node at a rate proportional to their frequency in blood, and then individual T cells travel through the lymph node exiting after time 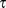. We model the different transit times 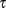 as a discrete series of 2 hour time intervals (i.e. 0,2,4,6 …). The proportion of T cells which spend time 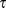 within the lymph node before exiting is denoted by 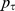. Our objective is to obtain a best estimate of the distribution p. The proportion of cells in the blood at time *t*, the input, is denoted by 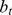. The proportion of cells exiting in the efferent lymphatic at time *t*, the output, is denoted by 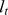. 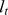 will then be proportional to a weighted sum of 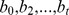, with the weights given by the proportion of cells with transit times 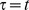, …, 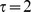, 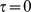 (denoted by the vector 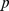). Thus, the percentage of labeled lymphocytes in efferent lymph can be expressed concisely in matrix notation as:
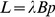
(5)where



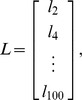


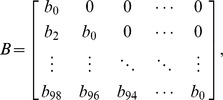


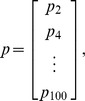


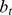
 is the percentage of labeled lymphocytes in blood at time t hours, 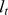 is the percentage of labeled lymphocytes in efferent lymph at time t hours, 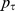 is the probability of naive T lymphocyte migration in the LN taking time 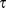 hours, and 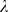 is the constant of proportionality. Thus, 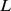 and 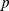 are vectors containing 50 entries and 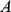 is a matrix of size 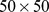. We collected data at high frequency at earlier time points in each experiment, when labelled cell numbers were changing very rapidly, and larger intervals (generally at 8 hour intervals) subsequently. We chose two hour intervals (i.e. 50 points per 100 hour experiment) as a balance between being able to capture the variable dynamics during the initial stages of each experiment, while avoiding over-interpolation during the latter stages.

The least absolute shrinkage and selection operator (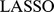) is a regularized form of ordinary least squares regression, regularized in the sense that the 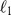 norm of the solution vector (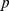 in our case) is constrained to be no greater than a user-defined constant. Specifically, we minimise
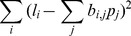
(6)subject to



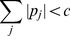
(7)The 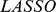 is of particular use for our study as it offers a natural way of ensuring our solution vector is a legitimate probability distribution (i.e. by choosing 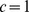). We also constrain 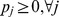 for the same reason. For a full discussion on the 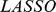 method, see [28]–[30] amongst many others.

Each distribution was initially found with no constraint placed on the solution vector, and the 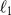 norm of the solution vector gave the best estimate for the value of 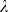 in each individual sheep. The system input matrix 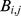 for each individual was then scaled by 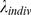. The solution vector 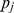 was then found with the constraint 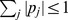 in place. All calculations were carried out using the 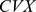 package in Matlab [31].

An extension of the 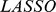 leads to the 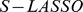, [32], and introduces a further term that penalises non-smooth solutions and modifies equation 6 to:
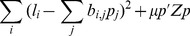
(8)where



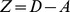
and



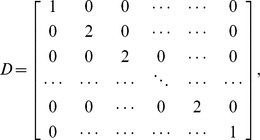


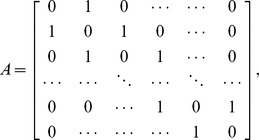
with *D* and *A* both matrices of size 50×t0. The constraint in equation (7) remains unchanged. The newly introduced parameter 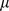 governs the extent of smoothing bias imposed on the solution. To determine a general distribution of migration times in the ovine LN, and to investigate possible improvements in accuracy of fit using the 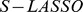, we formed a training set for each sample by concatenating randomly chosen data sets. For 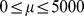, the corresponding 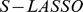 solution on the training set was found, and the distribution tested on a test set consisting of the remaining data sets. The optimal value of 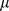 for each sample was given by the minimal RSS on the test set, providing a means of comparing the effects of concatenating training sets of various sizes, as well as obtaining an optimal distribution of migration times in the ovine LN.

To calculate 95% CIs, sample standard deviation for each interval was estimated using 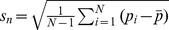, where 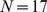, 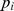 is the probablity of migration occurring in an individual (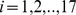) in each two-hour interval obtained from the 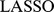 analysis, and 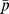 is the mean of the probabilities on each interval. 95% CIs are then given by 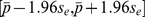, where 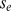 is the standard error of the mean given by 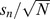.

#### Discrete random walk model

We explored the properties of a simplified, stochastic, discrete one-dimensional random walk model and optimised its parameters to fit the same set of experimental data. The model can be represented by a Markov Chain, whose transition state diagram is shown in fig. 4a. Lymphocytes are modelled as moving through the lymph node by moving from vertex 1 to vertex *N*, in *k* steps. In order to fit the experimental data to this model, we fix each step *k* to represent a time interval *dt*. The transition matrix *P* for this model is:
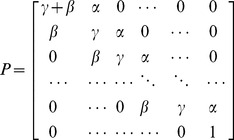
(9)


The probability of transition from vertex 1,(which models the high endothelial venules in the LN),to *N*, (which models the efferent lymphatics in the LN) in *k* steps (the first passage time) is given by the entry 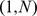 in 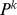. The behaviour of the system is determined by four parameters; two probabilities (the third is then fixed since they must sum to 1), the duration each time step represents in minutes, 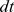, and the total number of vertices, 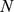. The probability of transition from vertex 1 to *N* occurring in exactly *ndt* minutes is given by 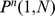. Since *dt* has units of minutes, and the migration data is analysed at 2 hour intervals, the probability 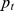 of migration occurring between time 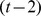, and time *t* hours is given by
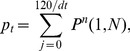
(10)where



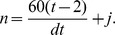



Again, we use *p*, along with *B*, to predict LN efflux (i.e. *L*) on a test set consisting of a random selection of nine individual sheep. The residual sum of squares (RSS) between the predicted efflux and actual efflux, *L*, is then used as a measure of goodness-of-fit for each distribution obtained from the stochastic model. We wish to determine the set of parameters 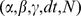 that best explain T lymphocyte migration times according to our data, i.e. that give the lowest RSS. We initially optimised over 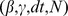 for 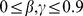 to 1 decimal place, 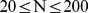 vertices and 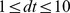 minutes. The optimal parameter set obtained from this analysis gave 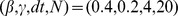. We then fixed 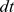 and *N* to these optimal values, and optimised 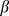 and 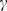 to 2 decimal places. Each pair of these parameter values gives a corresponding distribution of migration times, and predictions based on this corresponding distribution give a corresponding SSE over the test set.

#### Continuous distributions

We also investigated modelling the probabilities of different transit times by two different continuous distributions, the Gaussian distribution (two parameters) and the inverse Gaussian (two parameters). The inverse Gaussian was chosen because it has been shown to describe the probability distribution of first passage times for Brownian motion with drift [10]. The relevant parameters were optimised by varying the parameters, and using the resulting probability distribution to predict efflux (proportion of labelled cells in lymph) from input (proportion of labelled cells in blood), which is scaled by 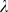, where 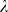 is found according to eqn. 5, on a training set of nine randomly chosen data sets. The parameters were chosen to minimise the SSE between predicted and actual efflux on the training set. The optimised distribution was then used on the remaining eight data sets, and SSE recorded.

## Supporting Information

Figure S1 **The predicted probability distribution of transit times for a representative sheep (R634) obtained using either linear or cubic spline interpolation.**
(TIF)
