# Supplementary figures and images for: Directional Migration of Recirculating Lymphocytes through Lymph Nodes via Random Walks
Source: PLoS One. 2012 Sep 20;7(9):e45262. doi: 10.1371/journal.pone.0045262 (PMC3447944; doi:10.1371/journal.pone.0045262)

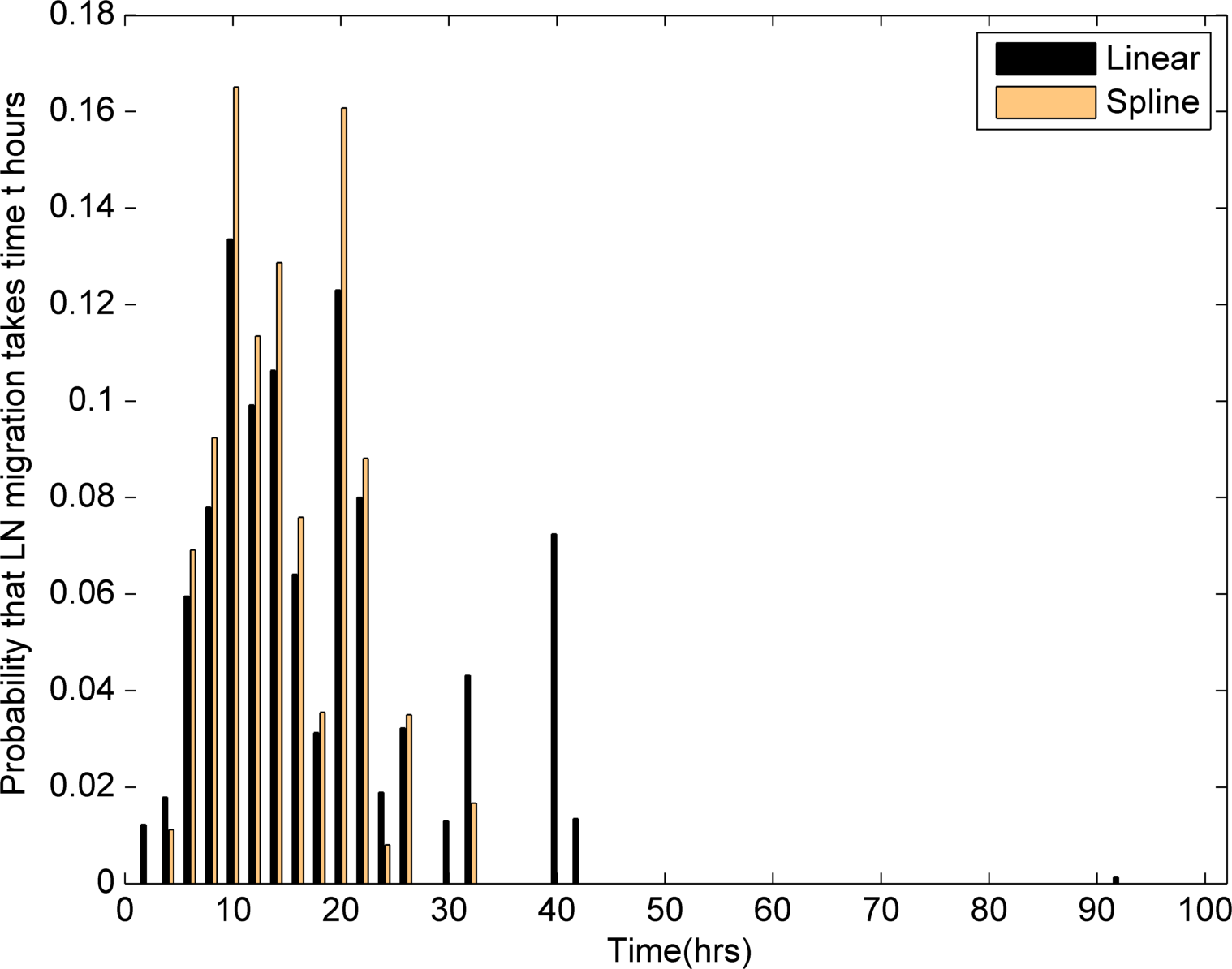

Supplement: Figure S1 — The predicted probability distribution of transit times for a representative sheep (R634) obtained using either linear or cubic spline interpolation. (TIF) [file pone.0045262.s001.tif]
